# Supplementary material for: Bayesian refinement of protein structures and ensembles against SAXS data using molecular dynamics
Source: PLoS Comput Biol. 2017 Oct 18;13(10):e1005800. doi: 10.1371/journal.pcbi.1005800 (PMC5662244; doi:10.1371/journal.pcbi.1005800)
Supplement: S1 Archive — (BZ2) [file pcbi.1005800.s011.tar.bz2 › gromacs_4.62_bayesian_saxs_refi/share/html/online/ngmx.html]

ngmx


| ngmx Main Table of Contents |

**VERSION 4.5  
Thu 26 Aug 2010**

---

### Description

ngmx is the Gromacs trajectory viewer. This program reads a
trajectory file, a run input file and an index file and plots a
3D structure of your molecule on your standard X Window
screen. No need for a high end graphics workstation, it even
works on Monochrome screens.

The following features have been implemented:
3D view, rotation, translation and scaling of your molecule(s),
labels on atoms, animation of trajectories,
hardcopy in PostScript format, user defined atom-filters
runs on MIT-X (real X), open windows and motif,
user friendly menus, option to remove periodicity, option to
show computational box.

Some of the more common X command line options can be used:  
-bg, -fg change colors, -font fontname, changes the font.

### Files

| option | filename | type | description |
| --- | --- | --- | --- |
| **-f** | traj.xtc | Input | Trajectory: xtc trr trj gro g96 pdb cpt |
| **-s** | topol.tpr | Input | Run input file: tpr tpb tpa |
| **-n** | index.ndx | Input, Opt. | Index file |

### Other options

| option | type | default | description |
| --- | --- | --- | --- |
| **-[no]h** | gmx\_bool | no | Print help info and quit || **-[no]version** | gmx\_bool | no | Print version info and quit || **-nice** | int | 0 | Set the nicelevel || **-b** | time | 0 | First frame (ps) to read from trajectory || **-e** | time | 0 | Last frame (ps) to read from trajectory || **-dt** | time | 0 | Only use frame when t MOD dt = first time (ps) |

### Known problems

- Balls option does not work- Some times dumps core without a good reason

---

http://www.gromacs.org  
gromacs@gromacs.org
